# Induction of Autopolyploidy and Preliminary Investigation of the Dwarfing Mechanism in *Hedychium coccineum*

**DOI:** 10.3390/plants14233573

**Published:** 2025-11-22

**Authors:** Fang Wang, Feixuan Jin, Xuanguo Liang, Jiangming Qiu, Qing Wang, Yunyi Yu, Rangcai Yu, Yanping Fan

**Affiliations:** 1 The Research Center for Ornamental Plants, College of Horticulture, South China Agricultural University, Guangzhou 510642, China; 2 College of Life Sciences, South China Agricultural University, Guangzhou 510642, China

**Keywords:** *Hedychium coccineum*, polyploid breeding, colchicine, dwarfism

## Abstract

In this study, *Hedychium coccineum* tetraploid plants and octaploid plants induced by colchicine were used as materials. The ploidy levels were precisely identified by combining root tip squash and flow cytometry analyses, and the differences between plants of different ploidy levels were systematically investigated at cytological, morphological, and molecular levels. The results showed that the highest polyploid induction efficiency was achieved when callus tissues were treated with 0.1 g/L colchicine for 4 days. The fluorescence peak value of the induced plants was twice that of the tetraploids, confirming their octaploid status. Compared with tetraploids, octaploid plants exhibited almost no apparent dormancy period, significantly slower growth, earlier flowering, and notably smaller inflorescences. Morphologically, they showed a dwarf phenotype characterized by narrower and lighter-colored leaves, fewer leaves per shoot, shorter internodes, and wider leaf angles, along with enhanced stress tolerance. Cytological observation revealed that cell area in internode tissues at the bud and seedling stages was generally larger in tetraploids than in octaploids, suggesting a reduction in cell size following genome duplication. Furthermore, transcriptome comparison between tetraploids and octaploids identified *HcPCNA1* as a candidate gene closely associated with plant height. Functional validation showed that overexpression of *HcPCNA1* in *Arabidopsis thaliana* significantly increased plant height, whereas silencing of *HcPCNA1* in *H*. *coccineum* via Virus-induced gene silencing (VIGS) resulted in a distinct dwarf phenotype with smaller leaves. Cytological and molecular evidence together indicate that *HcPCNA1* may influence plant height in *H. coccineum* through its role in promoting cell division and elongation. This finding provides new insights into the molecular mechanisms underlying plant architecture development in polyploid species.

## 1. Introduction

*Hedychium coccineum* is a perennial herbaceous ornamental plant belonging to the genus *Hedychium* in the family Zingiberaceae. It produces numerous, densely arranged flowers with unique shapes and vibrant colors, conferring high ornamental and landscape value [1]. However, its tall plant stature—reaching up to 2.5 m—makes it unsuitable for pot cultivation, which greatly limits its application in flower borders, landscaping, and hedges and consequently restricts its commercial development [2]. Moreover, frequent outbreaks of bacterial wilt of *Hedychium* in recent years have severely affected yields and hindered large-scale cultivation [3]. Dwarf breeding has become one of the major strategies in modern plant improvement, and numerous dwarf varieties with desirable agronomic traits have been successfully developed [4]. Dwarf cultivars are easier to manage and exhibit greater stability and productivity compared with tall types. For instance, in banana production, tall varieties are highly susceptible to wind damage, whereas dwarf cultivars can effectively reduce typhoon-related losses and offer advantages such as lower maintenance requirements and easier management [5]. Therefore, improving plant architecture, developing dwarf *H. coccineum* cultivars, and enhancing their stress tolerance represent effective approaches to accelerate the genetic improvement and industrial utilization of this species.

In ornamental plant breeding, dwarf cultivars have attracted considerable attention due to their suitability for small-scale and high-density planting, making them ideal breeding targets. Such plants are not only easier to manage with respect to flower shaping and pest control, but also significantly improve cultivation efficiency and management practices. Currently, plant breeding employs various approaches, including ploidy manipulation, mutation breeding, hybridization, and genetic engineering. Among these, ploidy breeding induces polyploid plants by altering the number of chromosomes in plant cells, thereby accelerating the development of varieties with desired traits [6]. Polyploid plants generally contain three or more complete sets of chromosomes and represent a form of environmental adaptation evolved by higher plants over long periods. Compared with diploids, polyploids often exhibit increased cell size, vigorous organ development, enhanced metabolic activity, stronger stress tolerance, and more stable genetic characteristics [7,8]. In recent years, with the advancement of molecular biology and cytogenetic techniques, research on plant polyploidy has received increasing attention. Studies have shown that polyploidization can induce significant changes in plant growth and development, morphological structure, and physiological and biochemical traits, generating new germplasm resources with breeding potential [9]. For example, ploidy-induced poplar plants display enlarged leaves and accelerated growth [10], whereas chromosome-doubled apple plants often exhibit slower growth and a dwarf phenotype [11].

In nature, plant polyploidy can arise through spontaneous mutations, but the mutation rate is typically only around 0.3%, which is insufficient to meet the large-scale demand for polyploid materials in research and breeding programs [12]. To enhance the efficiency of polyploid induction and shorten the breeding cycle, researchers commonly employ chemical mutagens (e.g., colchicine, sodium azide, oryzalin) or physical treatments (e.g., heat, cold, radiation) to promote chromosome doubling, thereby accelerating the development and selection of new polyploid lines [13]. Among these, colchicine is considered the most widely used and effective chemical inducer. It inhibits spindle formation during cell division, preventing normal chromosome segregation and thereby inducing chromosome doubling. Due to its ease of application, broad suitability, and high chromosome-doubling efficiency across multiple plant species, colchicine is recognized as one of the most reliable methods for obtaining polyploid plants. To date, researchers have successfully induced stable polyploid plants using colchicine in a variety of species, including apple (*Malus × domestica*) [11], *Ribes nigrum* L. [14], and pear (*Pyrus bretschneideri*) [13], demonstrating its broad applicability and high efficiency in polyploid breeding.

The morphogenesis of plant organs relies on precise cellular-level regulation and results from the coordinated action of multiple developmental processes [15]. The development and final form of lateral organs are primarily governed by the interplay between cell proliferation and cell expansion [16]. Cell proliferation controls the number of cells by modulating the rate and duration of cell cycle progression, thereby influencing the overall growth rate of organs. In contrast, cell expansion largely depends on the rate and duration of expansion, which are finely regulated by various physiological processes, including cell wall synthesis and remodeling, turgor pressure dynamics, and cytoskeletal rearrangements [17]. During plant growth and development, the precise regulation of cell proliferation is critical for organ morphogenesis and requires the coordinated action of cell cycle–related genes. Among these, Proliferating cell nuclear antigen (PCNA) serves as a key marker of cell division activity and plays a central role in DNA replication, repair, and cell cycle regulation [18]. As a cofactor of DNA polymerase δ, PCNA stabilizes the replication complex and facilitates DNA strand elongation, ensuring proper progression of the cell cycle. Beyond maintaining cell proliferation, PCNA also serves as an important molecular indicator of plant growth vigor and developmental status. Its expression levels are closely associated with plant growth rate, organ size, and the formation of dwarf phenotypes, providing valuable insights into the molecular mechanisms underlying plant morphogenesis [19].

Seed production in *Hedychium* species is generally low, and propagation relies predominantly on vegetative methods. However, long-term asexual propagation limits genetic recombination and trait innovation, often leading to variety degeneration and reduced genetic diversity. Research on ploidy breeding in *Hedychium* has demonstrated its potential to overcome these limitations. For instance, Hong et al. [2] successfully obtained tetraploid plants from diploid *Hedychium coronarium* via colchicine treatment, and the tetraploids exhibited significantly superior traits in plant height, leaf length and width, and flowering time compared with diploids, indicating that ploidy manipulation can markedly improve growth and developmental characteristics. Therefore, ploidy breeding, as an innovative genetic improvement approach, can provide new strategies and technical support for the selection of superior *Hedychium* cultivars by analyzing phenotypic variations induced by changes in ploidy. In the present study, tetraploid and colchicine-induced polyploid *H. coccineum* plants were used as materials. Ploidy levels were determined using root-tip squashes and flow cytometry, and the dwarfing mechanism of *H. coccineum* was systematically analyzed from morphological, cytological, and physiological perspectives. The results provide a theoretical basis and reference for polyploid dwarf breeding in *Hedychium* and offer valuable guidance for genetic improvement and the development of new cultivars.

## 2. Materials and Methods

### 2.1. Plant Material

The experimental plots were established at the floriculture cultivation base of South China Agricultural University, using tetraploid *H. coccineum* plants cultivated under natural conditions. Filaments, anthers, and young leaves were collected as explant materials for callus induction. *Arabidopsis thaliana* plants were grown in a growth chamber at 25 °C under a 16 h light/8 h dark photoperiod with a light intensity of 200 μmol m^−2^ s^−1^.

### 2.2. Colchicine Induction

Once sufficient callus had formed, it was transferred to proliferation medium for subculture at monthly intervals. Actively growing and uniform *H. coccineum* calli were inoculated into liquid media containing 0.05, 0.1, or 0.2 g/L colchicine, with colchicine-free medium serving as the control. Each treatment included 10 flasks, with 4 callus pieces per flask, and cultures were maintained on a rotary shaker at 90 rpm for 3~5 days in a growth chamber at 27 ± 2 °C. Following colchicine treatment, calli were transferred to colchicine-free proliferation medium for two subculture cycles, and subsequently moved to rooting medium for further development. The number of surviving calli and the total callus biomass in each flask were recorded before and after culture. Regenerated plants exhibiting morphological characteristics indicative of polyploidy were further subjected to chromosome analyses. Polyploid induction rate, callus proliferation fold, and survival rate were subsequently calculated.

Polyploid induction rate (%) = (Number of polyploid plants/Total number of regenerated plants) × 100%

Callus proliferation fold = Weight of callus after 30 days/Initial inoculum weight

Survival rate (%) = (Number of viable explants/Total number of inoculated explants) × 100%

### 2.3. Polyploid Identification of H. coccineum

The procedure was modified from Cotias-de-Oliveira [20]. Root tips (approximately 1~2 cm) were collected from tetraploid and colchicine-treated *H. coccineum* seedlings and placed in centrifuge tubes containing 0.1% colchicine solution for pretreatment at 4 °C for 8 h. After pretreatment, root tips were rinsed and fixed in Carnoy’s solution (ethanol: glacial acetic acid, 3:1) for 20 h. Fixed root tips were then washed with distilled water for 10~15 min, hydrolyzed in 1 M HCl at 60 °C for 10 min, and subsequently rinsed thoroughly with deionized water for 10~20 min. The root tips were then squashed on slides, and chromosome numbers were observed under a microscope (Zeiss, Oberkochen, Germany).

Ploidy analysis was performed on young leaves of tetraploid and colchicine-induced *H. coccineum* seedlings at the seedling stage, with 10 pseudostems randomly selected per group, using a CytoFLEX flow cytometer (Beckman Coulter, Brea, CA, USA). Four different nuclear isolation buffers—Galbraith, LB01, Otto, and Tris-MgCl_2_—were tested, and parameters including mechanical disruption intensity, centrifugation speed and duration, and staining time were systematically optimized. Through these experiments, optimal conditions were determined as follows: Otto buffer for nuclear isolation, centrifugation at 1000 rpm for 5 min, and staining for 10 min. Approximately 50 mg of young leaves was excised, placed in a Petri dish, and chopped finely, followed by the addition of 2 mL Otto I buffer. The mixture was incubated on ice for 10 min and filtered through a 400-mesh nylon sieve (approximately 35 µm pore size) into a 2 mL centrifuge tube. Nuclei were pelleted by centrifugation at 1000 rpm for 5 min at 4 °C, and the supernatant discarded. The pellet was resuspended in twice its volume of Otto II buffer, mixed thoroughly to obtain a nuclear suspension, and kept on ice. Propidium iodide (PI) and RNase solutions were then added to a final concentration of 50 μg/mL, and the suspension was incubated on ice in the dark for 10 min before analysis. The mean G1 peak fluorescence was calculated using CytExpert 2.4 software, and the ploidy level of each sample was determined by comparing the G1 peak fluorescence of tetraploid and induced plants.

### 2.4. Growth, Developmental Traits, and Morphological Measurements of Polyploid Plants

During the growth and development of *H. coccineum*, a comprehensive analysis of developmental traits was conducted, including observations of the vegetative growth phase, budding stage, dormancy period, flowering stage, inflorescence flowering period, single flower anthesis, and leaf emergence rate. During the flowering stage, tetraploid and octoploid plants were randomly sampled, and 35 quantitative traits—including plant height, canopy width, leaf length and width, inflorescence length and width, flower length and width, and bract dimensions—were measured using a vernier caliper (mm) and a ruler (cm). Each value represents the mean of three independent biological replicates, obtained from three different plants, to ensure accuracy and reliability.

### 2.5. Cytological Observation of Polyploid Plants

The aerial parts of tetraploid and octoploid *H. coccineum* plants were collected at the budding and seedling stages. After removing the outer leaf sheaths, tissues were excised from the lower internodes of the stem, and thin transverse sections of appropriate size were obtained and fixed in FAA solution (70% ethanol: glacial acetic acid: 38% formaldehyde = 90:5:5). The samples then underwent a standard paraffin-embedding workflow, including dehydration, clearing, wax infiltration, embedding, and sectioning. The resulting paraffin sections were stained with a safranin-fast green combination to enhance visualization of the anatomical structures [21,22]. Finally, the stained sections were examined under a microscope (Zeiss, Oberkochen, Germany), and the cell area within each field of view was measured and statistically analyzed using Motic Images 2000 software to obtain the average cell size.

### 2.6. RNA Extraction and Transcriptome Sequencing

Twelve tetraploid and twelve octoploid *H. coccineum* plants at the seedling stage were selected, and shoot apices were collected as experimental materials. Every three plants were pooled as one sample, with four biological replicates. Total RNA was extracted using the Plant Total RNA Extraction Kit (Magen, Guangzhou, Guangdong, China). RNA integrity was assessed via 0.8% agarose gel electrophoresis, while RNA concentration and purity were determined using a NanoDrop spectrophotometer (MIULAB, Hangzhou, Zhejiang, China) to ensure sample quality and integrity. Transcriptome sequencing was performed by Biomarker Technologies (Beijing, China) using the Illumina high-throughput sequencing platform. Sequencing of the constructed cDNA libraries generated a large amount of high-quality raw data. Subsequently, stringent quality control was performed to ensure the reliability of the downstream analyses. The raw sequencing data were filtered by removing reads in which more than 20% of the bases had a quality score below 20 or in which the proportion of ambiguous bases (N) exceeded 5%. In addition, rRNA sequences were removed. The remaining high-quality reads (clean data) were retained for subsequent analyses.

Transcript abundance was normalized using the RPKM. Differentially expressed genes (DEGs) were identified using the DESeq2 package in R software (version 4.3.1), with the screening criteria set as |Fold change| ≥ 2 and FDR < 0.01. The identified DEGs were annotated by comparison against multiple public databases, including COG, GO, KEGG, KOG, Pfam, Swiss-Prot, eggNOG, and NR, to obtain comprehensive functional information and to summarize the number of genes annotated in each database. GO and KEGG analyses were subsequently performed to explore the biological functions and metabolic pathways significantly enriched among the DEGs.

### 2.7. Cloning, Sequence Alignment, and Phylogenetic Analysis of HcPCNA1

The full-length coding sequence of *HcPCNA1* was amplified by PCR using gene-specific primers (Supplementary Table S1). Homologous PCNA amino acid sequences from multiple species were obtained from the National Center for Biotechnology Information (NCBI) database. Multiple sequence alignment was performed using DNAMAN 7.0, and a phylogenetic tree was constructed using MEGA 11 software.

### 2.8. Quantitative Real-Time PCR (qRT-PCR) Analysis

Total RNA was extracted using the Plant Total RNA Extraction Kit (Magen), and first-strand cDNA was synthesized using the Evo M-MLV Premix Kit. qRT-PCR was performed on an ABI 7500 Real-Time PCR System. Each 20 μL reaction contained 0.4 μL of 10 μM forward primer, 0.4 μL of 10 μM reverse primer, 2 μL of cDNA template, 7.2 μL of qPCR SYBR Green Mix (Yeasen, Shanghai, China), and 10 μL of ddH_2_O. The amplification program was as follows: pre-denaturation at 95 °C for 5 min; denaturation at 95 °C for 10 s; annealing at 55 °C for 20 s; and extension at 72 °C for 30 s, for a total of 40 cycles. ddH_2_O was used as a negative control. Each sample was analyzed in at least three biological replicates, and relative gene expression levels were calculated using the 2^−ΔΔCt^ method. The primer sequences used for target genes and internal reference genes are listed in Supplementary Table S1.

### 2.9. Arabidopsis thaliana Genetic Transformation

The pOx-*HcPCNA1* construct was generated using a double restriction enzyme digestion. The recombinant plasmid was subsequently introduced into *Agrobacterium tumefaciens* strain GV3101 via the freeze–thaw transformation method and then transferred into *Arabidopsis thaliana* (wild-type, WT) using the floral-dip procedure [23]. Transgenic plants were selected on medium containing kanamycin (25 mg/mL), and positive lines were verified by PCR. Homozygous T2 plants were identified and used for subsequent analyses. Primer sequences are provided in Supplementary Table S1. Plant height (cm) was measured at the late developmental stage of *Arabidopsis*. Expression of the *HcPCNA1* gene was quantified using *Arabidopsis thaliana* Actin as the internal reference gene (Supplementary Table S1), and relative expression levels were calculated using the 2^−ΔΔCt^ method.

### 2.10. Virus-Induced Gene Silencing (VIGS) of HcPCNA1

Primers with adapters were designed in the specific regions at the 5′ or 3′ end of the HcPCNA1 gene sequence to amplify a 200 bp fragment. The target fragment was then cloned into the pTRV2 vector to construct a VIGS vector. The recombinant pTRV2-HcPCNA1 and pTRV1 plasmids were transformed into *A. tumefaciens* strain EHA105. Positive colonies were cultured until the bacterial suspension reached an OD_600_ of 1.0~1.2, then resuspended in MES buffer to an OD_600_ of ~0.8 and incubated in the dark at 28 °C for 3 h. Equal volumes of pTRV1 and pTRV2-HcPCNA1 cultures were mixed, and in vitro-grown *Hedychium* seedlings were inverted and submerged in the bacterial suspension. Vacuum infiltration was applied at 0.8 kPa for 15 min. Successful infiltration was indicated by deep green coloration of stems and leaves. Seedlings were then blotted to remove excess bacteria and incubated at ~25 °C. Subsequently, phenotypic changes, including plant height, were recorded. RNA was extracted from the internode regions of silenced plants, and relative expression levels of HcPCNA1 and related genes were quantified by qRT-PCR using HcGAPDH as the internal reference.

### 2.11. Data Processing

Data were organized using Microsoft Excel 2010 and statistically analyzed with SPSS 26.0 software. One-way ANOVA analysis was performed to evaluate differences among traits, and significant differences between means were determined using the LSD test. All results are presented as mean ± standard deviation (SD).

## 3. Results

### 3.1. Effects of Colchicine Treatment Conditions on Polyploid Induction Efficiency and Tissue Growth in H. coccineum

**Figure 1:**
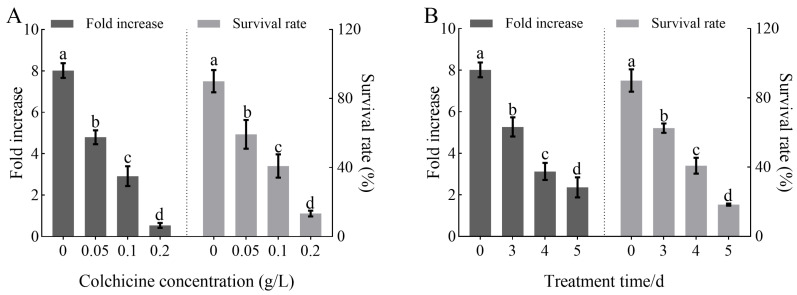
Effects of colchicine concentration and treatment duration on the growth of primary calli in *Hedychium coccineum*. (**A**) Proliferation fold and survival rate of primary callus under different colchicine concentration treatments; (**B**) Proliferation fold and survival rate of primary callus under different colchicine treatment durations. Note: Different lowercase letters indicate significant differences (*P* < 0.05) in primary callus under various treatment conditions.

The results of colchicine treatments on *H. coronarium* callus indicate that both colchicine concentration and exposure duration significantly affect callus growth and viability. As shown in Figure 1A, increasing colchicine concentrations led to a significant reduction in both proliferation fold and survival rate of the callus (*p* < 0.05). Notably, at 0.2 g/L, callus growth and viability were markedly inhibited, suggesting a high sensitivity of the callus to colchicine concentration. Figure 1B demonstrates that prolonged exposure also resulted in a significant decline in proliferation and survival rates (*p* < 0.05). According to Table 1, the interaction between colchicine concentration and treatment duration exerted a significant effect on the growth and viability of primary callus. Overall, increasing colchicine concentration combined with longer treatment duration caused a continuous and significant decrease in both proliferation fold and survival (*p* < 0.05). When the concentration reached 0.2 g/L and treatment lasted for 5 days, the callus completely lost viability and died (see Supplementary Figure S1). In summary, both proliferation fold and survival rates of the callus decreased significantly with increasing colchicine concentration and treatment duration (Figure 1 and Table 1).

**Figure 2:**
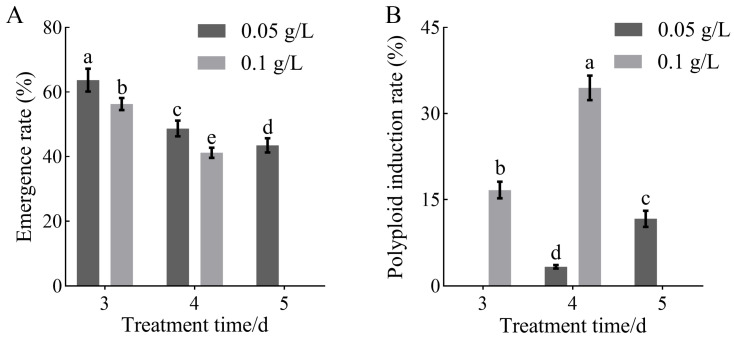
Effects of colchicine treatment on the seedling emergence rate and polyploid induction rate of *H. coccineum.* (**A**) Emergence rate of *H. coccineum* under different colchicine concentrations and treatment durations. (**B**) Polyploid induction rate of *H. coccineum* under different colchicine concentrations and treatment durations. Note: Different lowercase letters indicate significant differences among treatments at *p* < 0.05.

In addition, observation of colchicine-treated calli of *H. coccineum* revealed a clear dose–time-dependent relationship between seedling emergence rate and polyploid induction efficiency. Under the same colchicine concentration, the seedling emergence rate gradually decreased with increasing treatment duration, whereas the polyploid induction rate significantly increased (Figure 2). Similarly, under the same treatment duration, when the colchicine concentration was below the lethal threshold, the polyploid induction rate increased with higher colchicine concentrations. Among all treatments, 0.1 g/L colchicine for 4 days resulted in the highest polyploid induction efficiency.

### 3.2. Chromosome Number and Ploidy Analysis of Mutagenized Materials of H. coccineum

**Figure 3:**
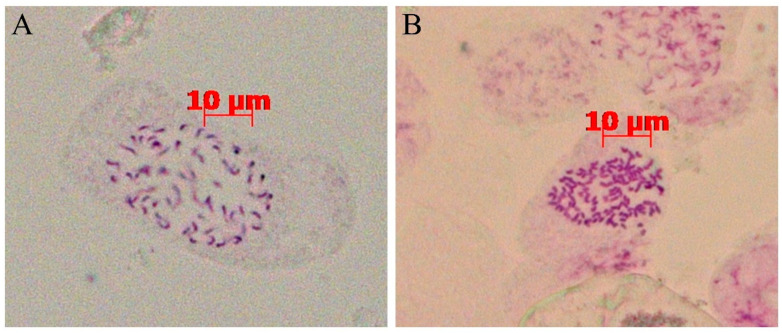
Chromosomal observations of tetraploid and colchicine-induced plants of *H coccineum.* (**A**) Tetraploid *H. coccineum* plant; (**B**) Colchicine-induced *H. coccineum* plant.

Chromosome counting was performed on root tip cells of both tetraploid and colchicine-treated *H. coccineum*. As shown in Figure 3, the wild-type plants possessed 68 chromosomes, confirming their autotetraploid nature, which is consistent with previous reports [24,25]. In contrast, the colchicine-treated mutants exhibited 136 chromosomes, indicating successful chromosome doubling and the formation of auto-octoploid plants.

**Figure 4:**
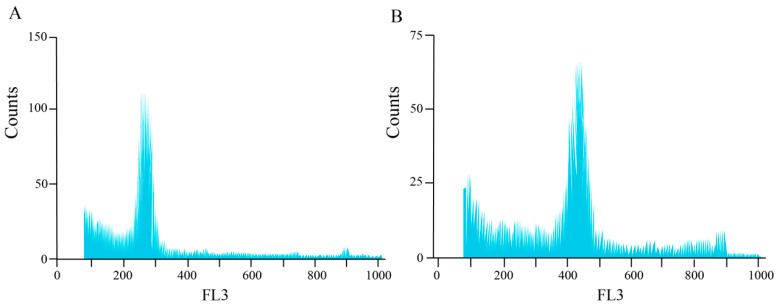
Flow cytometric analysis of tetraploid and colchicine-induced *H. coccineum* plants. (**A**) Tetraploid *H. coccineum* plant; (**B**) Colchicine-induced *H. coccineum* plant.

Furthermore, flow cytometric analysis was performed on both tetraploid and colchicine-induced *H. coccineum* plants to compare their nuclear DNA content and mean fluorescence intensity. The results are presented in Figure 4 and Table S2. The ratio of mean fluorescence intensity between induced plants and the tetraploids ranged from 1.71 to 2.20, with a low coefficient of variation (<8%). These findings indicate that most of the induced *H. coccineum* plants underwent successful chromosome doubling and are identified as octoploids.

### 3.3. Analysis of Growth and Developmental Characteristics and Morphological Traits of Tetraploid and Octoploid H. coccineum

**Figure 5:**
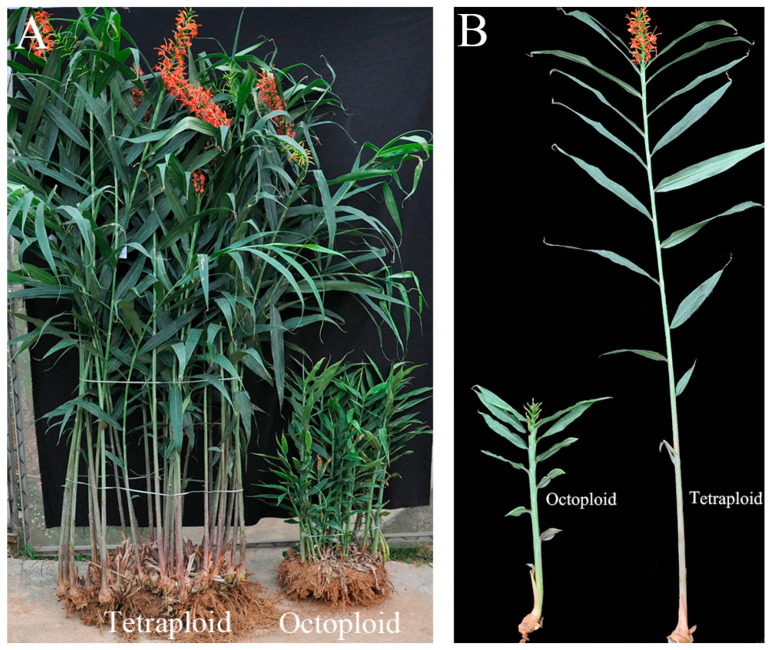
Phenotypes of tetraploid and octoploid *H. coccineum* plants. (**A**) Comparison of whole clumps of tetraploid and octoploid *H. coccineum* plants. (**B**) Comparison of pseudostems of tetraploid and octoploid *H. coccineum* plants.

Morphological traits of tetraploid and octoploid *H. coccineum* plants were systematically observed and compared (Figure 5). The results indicated that octoploid plants exhibited pronounced dwarfism, with markedly shortened internodes, smaller leaves, and overall weaker vigor. Statistical analysis of flowering traits revealed significant differences between the two ploidy levels in most phenotypic parameters (Table 2). Regarding the growth and developmental cycle, both tetraploids and octoploids displayed similar durations of the vegetative growth period and bud development stage, approximately 4~5 months and 2~3 months, respectively. However, notable differences were observed in dormancy characteristics: tetraploids exhibited a clear dormancy period of approximately 2 months per year, whereas octoploids rarely entered dormancy. The flowering period of tetraploids was relatively long, extending from early June to late October, with individual inflorescences lasting about 8~10 days and single flowers 2~3 days. In contrast, octoploids, affected by colchicine-induced phytotoxicity, showed a significantly shortened flowering period and reduced pollen viability. Moreover, leaf emergence in tetraploids occurred at a faster rate than in octoploids, indicating higher growth speed and greater plant vigor.

**Figure 6:**
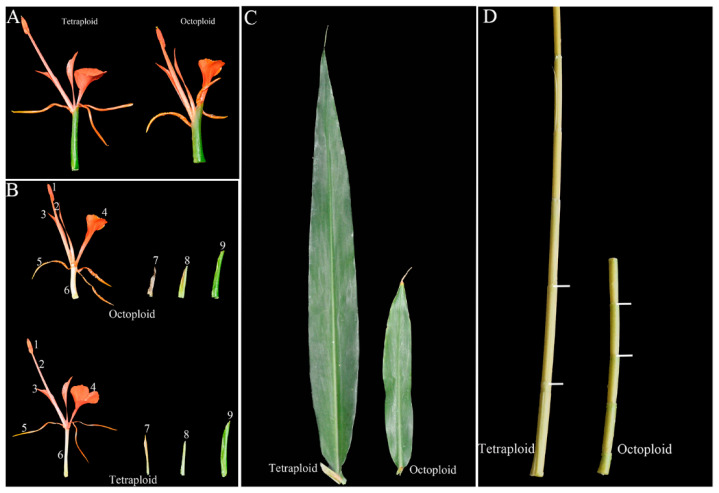
Phenotypes of different organs in tetraploid and octoploid *H. coccineum plants.* (**A**) Comparison of individual flowers; (**B**) Structure of a single flower; (**C**) Comparison of leaves; (**D**) Comparison of internode length. 1, Anther; 2, Filament; 3, Lateral petal; 4, Lip petal; 5, Petal; 6, Corolla tube; 7, Calyx; 8, Small bract; 9, Bract.

**Figure 7:**
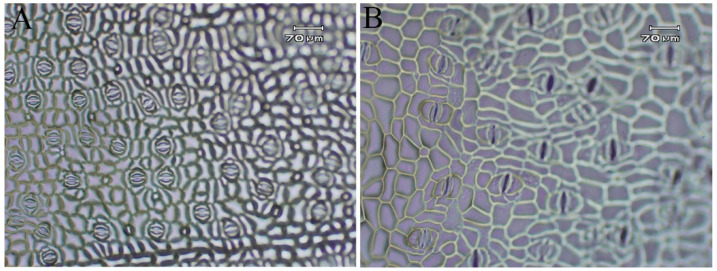
Stomata size and density in *H. coccineum* (scale bar = 70 μm). (**A**) Tetraploid; (**B**) Octoploid.

**Figure 8:**
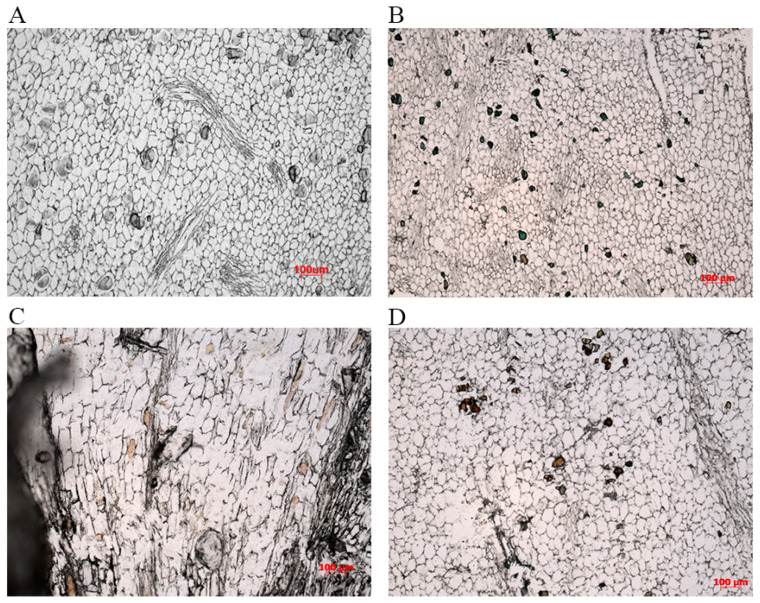
Observation of internodal cells in tetraploid and octoploid *H. coccineum* plants during the bud and seedling stages. (**A**) Internodal cells of tetraploid *H. coccineum* at the bud stage; (**B**) Internodal cells of octoploid *H. coccineum* at the bud stage; (**C**) Internodal cells of tetraploid *H. coccineum* at the seedling stage; (**D**) Internodal cells of octoploid *H. coccineum* at the seedling stage.

**Figure 9:**
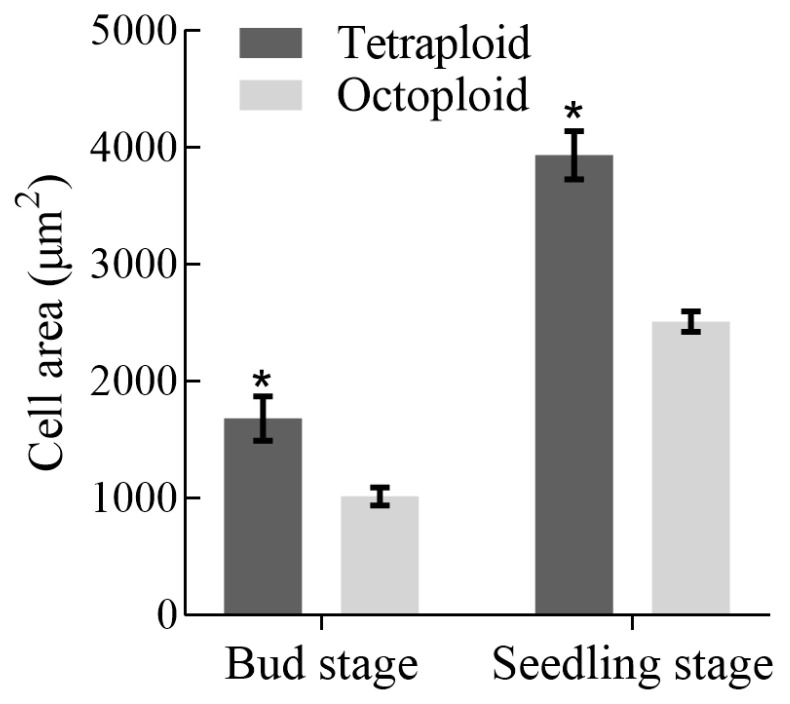
Size of internodal cells in tetraploid and octoploid *H. coccineum* plants at the bud and seedling stages. Note: * *p* < 0.05 indicates the significant difference in cell size between tetraploid and octoploid *H. coccineum* at the same developmental stage.

In terms of morphological traits, tetraploid *H. coccineum* plants exhibited significantly higher values than octoploids for numerous characteristics, including plant height, plant spread, number of internodes, average internode length, leaf length, leaf width, stomatal density, inflorescence length and width, number of bracteoles, flower length and width, bract length, corolla tube length, lateral petal width, pistil length, stamen length, and filament length. In contrast, octoploids showed significantly greater leaf angles, calyx width, corolla tube width, pistil width, anther length, and filament width compared with tetraploids. Moreover, tetraploid leaves displayed significantly higher stomatal density but reduced stomatal length and width relative to octoploids (Figure 6 and Figure 7 and Supplementary Figure S2). These results suggest that polyploidization may induce a trade-off between stomatal number and size during leaf development, where increased stomatal density is generally accompanied by reduced stomatal dimensions, potentially affecting gas exchange and transpiration efficiency. Furthermore, histological analysis of internodal tissues during the bud and seedling stages revealed that tetraploid cells had significantly larger cell areas than octoploids (Figure 8 and Figure 9), indicating that changes in ploidy level exert a pronounced effect on cell morphology. This effect may influence overall plant growth and development through alterations in cell expansion and division patterns.

### 3.4. Transcriptomic Analysis of Tetraploid and Octoploid H. coccineum Plants

**Figure 10:**
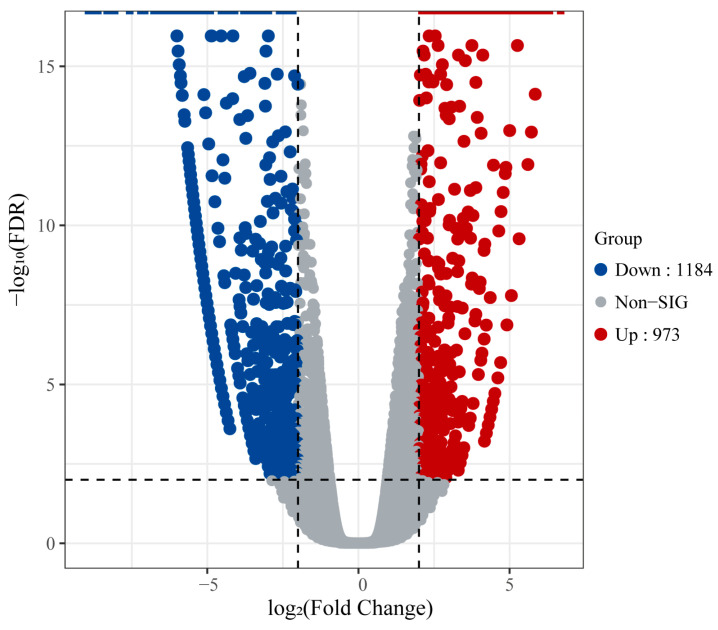
Volcano plot of differentially expressed genes in tetraploid and octoploid *H. coccineum*.

Transcriptome sequencing was performed on the shoot apices of tetraploid and octoploid *H. coccineum* seedlings, yielding a total of 78,334,397 clean reads. The GC content exceeded 49%, and the Q30 values were above 94%, indicating high sequencing quality and a low error rate. The overall mapping efficiency of the samples was greater than 68%, demonstrating the reliability of the sequencing data (Table 3). Differentially expressed genes (DEGs) between tetraploid and octoploid plants were identified using a threshold of |fold change| ≥ 2 and FDR < 0.01. A total of 2157 DEGs were detected, of which 973 genes were upregulated and 1184 genes were downregulated (Figure 10).

**Figure 11:**
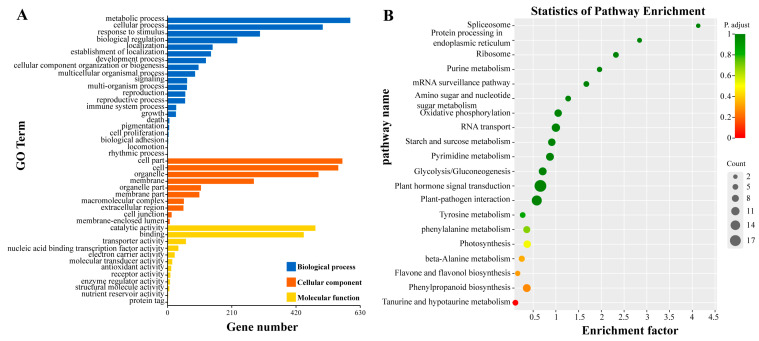
GO and KEGG enrichment analysis of differentially expressed genes in *H. coccineum.* (**A**) Bar chart of GO enrichment analysis of DEGs; (**B**) Bubble chart of KEGG enrichment analysis of DEGs.

The functions of DEGs were further explored using the Gene Ontology (GO) and Kyoto Encyclopedia of Genes and Genomes (KEGG) databases. GO annotation revealed that these DEGs were primarily enriched in three main categories: Biological Process (BP), Cellular Component (CC), and Molecular Function (MF). Within the BP category, DEGs were mainly associated with metabolic processes, cellular processes, and responses to stimuli. In the CC category, DEGs were predominantly related to cell parts, cells, and organelles. For the MF category, DEGs were mainly involved in catalytic activity, binding, and transporter activity (Figure 11A). KEGG enrichment analysis indicated that 271 DEGs were mapped to 88 pathways. The KEGG enrichment scatter plot showed that these DEGs were mainly enriched in the “plant hormone signal transduction” pathway (Figure 11B).

### 3.5. Functional Validation and Analysis of the Key Dwarfing Regulatory Gene HcPCNA1 in H. coccineum

#### 3.5.1. Gene Structure and Phylogenetic Analysis of HcPCNA1

**Figure 12:**
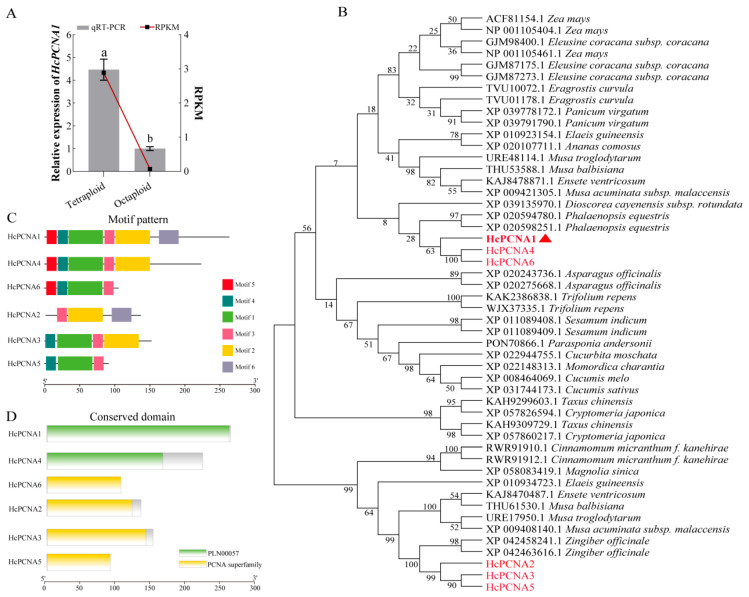
qRT-PCR and gene structure analysis of the *HcPCNA1* (**A**) qRT-PCR analysis and RPKM values of *HcPCNA1* in the stems of tetraploid and octoploid *H. coccineum*; (**B**) Phylogenetic analysis of HcPCNA and highly homologous proteins from other species in NCBI; (**C**) Conserved motif distribution of HcPCNA proteins; (**D**) Gene structure analysis of *HcPCNA*. Note: Different lowercase letters indicate significant differences among samples at *p* < 0.05; The red font represents all *HcPCNA* genes in *H. coccineum;* The red triangle represents the key gene *HcPCNA1*, which controls the dwarfing of *H. coccineum*.

In this study, a PCNA gene (T1_Unigene_BMK.68886) was identified and designated as *HcPCNA1*, which exhibited significantly higher expression in tetraploid plants compared to octoploids (Figure 12A). PCNA (proliferating cell nuclear antigen) is known to play a key role in DNA replication, repair, and cell cycle regulation [18]. The ORF of *HcPCNA1* is 450 bp in length, encoding 149 amino acids with a predicted molecular weight of 16.39 kDa. Using the amino acid sequence of *HcPCNA1*, homologous genes were screened from the *Hedychium* genome, resulting in 5 homologs designated as *HcPCNA2*, *HcPCNA3*, *HcPCNA4*, *HcPCNA5*, and *HcPCNA6*. To investigate the evolutionary relationships between *H. coccineum* PCNAs and PCNA homologs from other species, a BLAST search (https://blast.ncbi.nlm.nih.gov/Blast.cgi accessed on 22 October 2025) was conducted in NCBI GenBank, and 44 PCNA proteins from 25 different species were selected for phylogenetic analysis. Phylogenetic analysis revealed that HcPCNA1 shares a high degree of homology with PCNA proteins from *Phalaenopsis equestris* (Figure 12B). Conserved motif analysis of the six HcPCNA proteins indicated that HcPCNA1 contains three conserved motifs: Motif2, Motif3, and Motif6 (Figure 12C), and all six proteins were classified within the PCNA subfamily (Figure 12D).

#### 3.5.2. Overexpression and Virus-Induced Silencing of HcPCNA1 Significantly Affect Plant Growth

**Figure 13:**
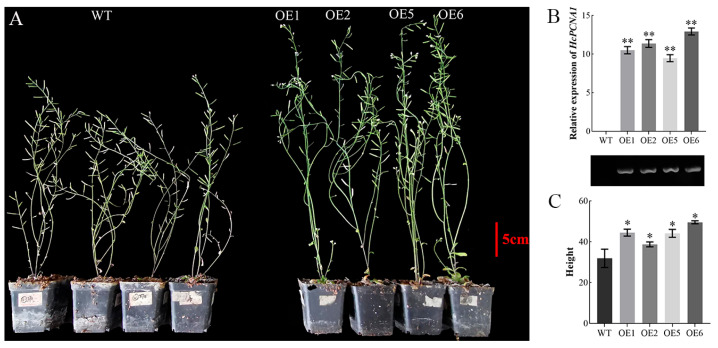
Phenotypes of *Arabidopsis thaliana* transgenic lines overexpressing *HcPCNA1.* (**A**) Phenotypes of wild-type(WT) and *HcNAC1* overexpression lines(OE) in *Arabidopsis*; (**B**) qRT-PCR analysis of *HcNAC1* in *Arabidopsis* wild-type and overexpression lines; (**C**) Plant height of wild-type and *HcNAC1* transgenic lines. Note: * *p* < 0.05, ** *p* < 0.01.

**Figure 14:**
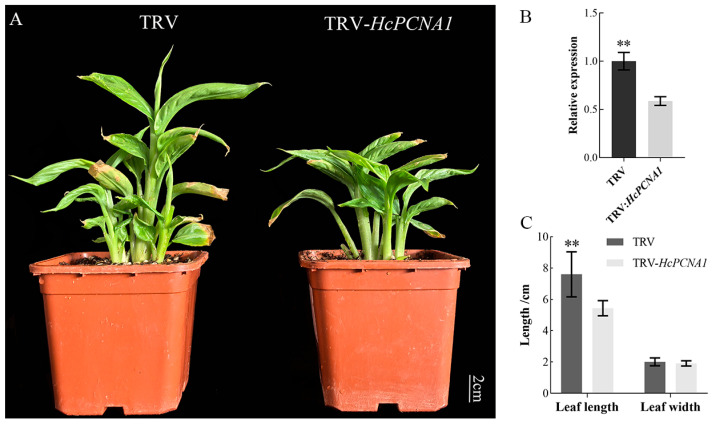
Phenotypes and gene expression of *H. coccineum* seedlings following virus-induced silencing of *HcPCNA1.* (**A**) Phenotype of *H. coccineum* after VIGS of the *HcNAC1* gene; (**B**) qRT-PCR analysis of *HcNAC1* in *H. coccineum* after VIGS of HcNAC1; (**C**) Leaf length and width of *H. coccineum* after VIGS of *HcNAC1*. Note: ** *p* < 0.01.

To investigate the biological function of *HcPCNA1*, transgenic *A. thaliana* plants overexpressing *HcPCNA1* (OE) were generated. PCR verification confirmed the successful establishment of independent *HcPCNA1* overexpression lines (Figure 13). Compared with the wild type (WT), four overexpression lines (OE-1/2/5/6) exhibited significantly increased plant height. In parallel, VIGS system using TRV1/TRV2 was employed to transiently silence *HcPCNA1* in *H. coccineum* tissue-cultured seedlings. *Agrobacterium* harboring the *HcPCNA1* silencing construct was applied via vacuum infiltration, and plants were cultured for 40 days before phenotypic assessment and qRT-PCR analysis. The results showed that silencing of *HcPCNA1* led to a reduction in seedling height and a significant decrease in leaf length compared with control plants. Furthermore, the relative expression level of *HcPCNA1* was markedly decreased in silenced seedlings (Figure 14).

**Figure 15:**
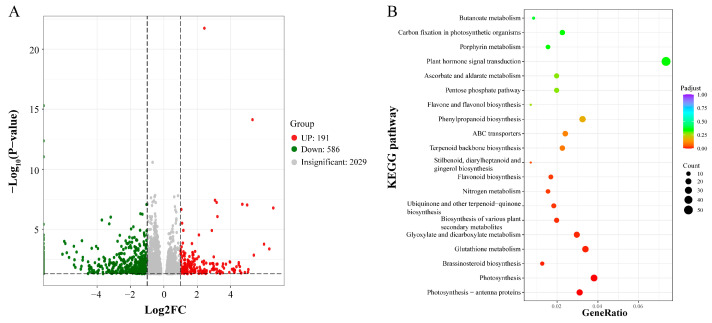
Analysis of differentially expressed genes between wild-type and *HcPCNA1*-silenced plants. (**A**) Volcano plot of DEGs; (**B**) Bubble plot of KEGG enrichment analysis of DEGs.

To investigate the regulatory mechanisms of *HcPCNA1*, transcriptome sequencing was performed on wild-type (WT) and TRV-*HcPCNA1 H. coccineum* plants. Compared with control plants (CK), a total of 191 upregulated and 586 downregulated differentially expressed DEGs were identified in *HcPCNA1*-silenced plants (Figure 15A). KEGG pathway enrichment analysis revealed that these DEGs were primarily associated with brassinosteroid biosynthesis, plant hormone signal transduction, phenylpropanoid biosynthesis, and ABC transporter pathways (Figure 15B).

**Figure 16:**
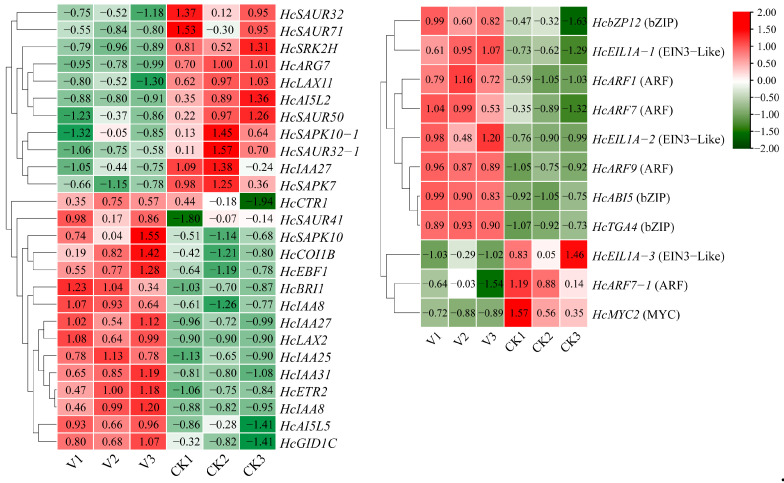
Differential gene expression in the plant hormone signal transduction pathway.

To further elucidate the molecular basis of dwarfism in *H. coccineum*, we analyzed the enrichment of DEGs within the plant hormone signal transduction pathway (Figure 16). A total of 37 DEGs were found to participate in this pathway, including key genes involved in responses to auxin, ethylene, and abscisic acid. Among these, 9 transcription factors were significantly upregulated and 3 were downregulated in TRV-*HcPCNA1* plants, suggesting that changes in transcription factor expression within hormone signaling pathways may play a crucial role in the formation of dwarf phenotypes. These DEGs likely regulate *HcPCNA1* expression directly or indirectly through the plant hormone signaling network. Previous studies have demonstrated that certain ARF, EIL, and bZIP family transcription factors mediate the transcriptional regulation of genes associated with cell division and elongation, thereby affecting cell proliferation and plant morphology [26,27,28]. Accordingly, it is plausible that these hormone-responsive transcription factors act in concert with *HcPCNA1* to modulate dwarfing in *H. coccineum*. Future studies will employ molecular biology approaches to validate the interactions between these transcription factors and *HcPCNA1*, systematically elucidate their protein–protein and transcriptional regulatory networks, and provide direct evidence for the molecular basis of dwarfism in *H. coccineum*.

## 4. Discussion

Colchicine is one of the most widely used and effective chemical mutagens for inducing polyploidy in plants. It primarily functions by inhibiting spindle formation, thereby preventing chromosome segregation during cell division and resulting in chromosome doubling within the nucleus [29]. Both the concentration and duration of colchicine treatment are critical factors influencing the efficiency of polyploid induction [30]. Studies in pear have shown that increasing colchicine concentration and extending treatment duration enhance the frequency of chromosome doubling; however, exceeding a certain threshold reduces the survival rate of polyploid plants [13]. In *Zingiber officinale*, treatment with 0.2% colchicine for 6 or 24 h achieved relatively high polyploid induction rates, reaching up to 50%, whereas increasing the concentration to 0.4% failed to produce polyploid plants [31]. Consistent with these findings, our study showed that treating *Hedychium callus* with 0.1 g/L colchicine for 4 days resulted in a high polyploid induction efficiency.

Morphological changes induced by colchicine treatment do not necessarily indicate successful chromosome doubling. While polyploid induction often results in observable morphological variation, the presence of such phenotypic changes alone cannot confirm polyploidy [32]. Root-tip chromosome squashes provide the most direct method for determining chromosome number, but this approach is time-consuming and often limited by the availability and quality of plant material, which can compromise observation results. In contrast, flow cytometry offers high accuracy, minimal sample requirements, and rapid detection, making it a key method for studying plant ploidy. By measuring DNA content within cells and analyzing the positions of fluorescence peaks, the ploidy level of plants can be reliably determined [33]. Currently, many researchers have combined root-tip chromosome squashes with flow cytometry to achieve more accurate determination of plant ploidy. This combined approach has been successfully applied in species such as *Cymbidium aloifolium* [34], *Cyclocarya paliurus* [35], tomato (*Solanum lycopersicum*) [36], and persimmon (*Diospyros kaki* Thunb.) [37]. In this study, the ploidy levels of tetraploid and mutant *H. coccineum* plants were determined using root-tip chromosome observation and flow cytometry. Flow cytometric analysis revealed that the mean fluorescence intensity of the mutant plants was 1.71–2.20 times that of the tetraploid plants, indicating successful chromosome doubling. Root-tip chromosome counts showed that tetraploid plants possessed 68 chromosomes (2n = 4x = 68), consistent with the results reported by Yu [25], whereas the mutant plants contained 136 chromosomes (2n = 8x = 136), confirming their octoploid status.

Changes in chromosome ploidy play a crucial role in plant evolution. Allopolyploids often exhibit superior traits compared with their diploid progenitors, including enhanced morphological variation, greater genetic adaptability, and increased stress tolerance [38]. Generally, chromosome doubling can substantially promote plant growth and development, leading to larger leaves and longer petioles, as well as enhanced metabolic activity. Consequently, polyploidization has considerable benefits in plant breeding and genetic improvement programs [39]. Chen et al. [39] reported that increased ploidy in *Raphanus sativus* is associated with longer and wider leaves as well as larger flowers. Consistently, in our study, octoploid *H. coccineum* plants exhibited greener pseudostems and larger leaf angles compared with tetraploid plants. Moreover, octoploid plants showed significant reductions in plant height, plant spread, leaf length, leaf width, internode length, and growth rate, while displaying enhanced cold tolerance and lodging resistance, a pattern similar to that observed in polyploid hybrid sweetgum [40]. The most pronounced morphological feature of octoploid *H. coccineum* was a clearly dwarfed plant stature, a phenomenon also reported in various other species. It has been observed that genome doubling often leads to growth inhibition or dwarfism. For example, increased ploidy levels in *Allium sativum* L. [38], *Ziziphus jujuba* [41], apple [11], and *Populus* [42] result in reduced plant height, slower growth rates, and shortened internodes. These findings suggest that genome doubling may alter overall plant growth and developmental patterns—potentially by affecting cell division rates and elongation capacity—thereby leading to the formation of dwarf phenotypes.

Polyploidization in plants is often accompanied by reduced growth rates, which may result from chromosome doubling causing a marked increase in cell size and a decrease in cell density. These cellular-level changes can alter tissue architecture and organ development, ultimately affecting the size of cells, flowers, and fruits [43]. Such alterations may inhibit cell elongation and division rates, thereby slowing overall growth [44]. In this study, internodal cells of octoploid *H. coccineum* during both the bud and seedling stages were significantly smaller than those of tetraploids, and leaf emergence rates were reduced. These observations suggest that cell division capacity and elongation in roots are suppressed during development, contributing to the dwarf phenotype. Additionally, growth and developmental characteristics of octoploid plants were compared with tetraploids, revealing that octoploid plants exhibit almost no dormancy and a markedly shortened flowering period. This may result from irregular chromosome segregation following genome doubling, where sister chromatids fail to separate during meiosis, leading to gamete chromosomal variation, reduced pollen viability, and a shortened flowering duration [45].

Plant dwarfism is often closely associated with cellular processes such as cell division and morphological changes. Wang et al. [19] reported that fiber cells in dwarf bamboo are significantly shorter, and that variations in cell size and morphology are closely linked to cell proliferation and expansion. Changes in cell proliferation largely depend on the activity of cell cycle–regulating genes, among which PCNA serves as a key factor, playing a central role in DNA replication and repair. During plant development, DNA in somatic cells is frequently exposed to various physical and chemical stresses, which can cause structural damage and severely impair normal cell proliferation and growth. PCNA facilitates the timely recognition of damaged sites, recruits repair proteins, and completes DNA repair, thereby maintaining cell division and growth activity [46,47]. In this study, we further explored the role of PCNA in regulating plant height. Overexpression of *HcPCNA1* in *A. thaliana* resulted in significantly increased plant height, indicating that elevated PCNA expression promotes cell proliferation and elongation. Conversely, VIGS of *HcPCNA1* in *H. coccineum* led to pronounced dwarfism, characterized by reduced overall height and shorter leaves. Coupled with prior observations of reduced cell size, these findings suggest that downregulation of *HcPCNA1* slows cell division and reduces cell area, thereby contributing to the dwarf phenotype. Collectively, these findings indicate that PCNA not only plays a central role in the cell cycle and DNA repair but also significantly influences plant height by regulating cell growth and organ elongation. It is noteworthy that in the polyploid materials examined in this study, the expression of *HcPCNA1* was downregulated in octoploid plants; however, the relationship between this transcriptional change and polyploidization remains complex. Polyploidization is often accompanied by gene dosage effects and alterations in chromosomal behavior, which may indirectly influence the transcription of certain cell cycle-related genes. Nevertheless, it is not yet possible to determine whether the reduced expression of *HcPCNA1* in octoploids is directly triggered by polyploidization itself. Therefore, we tend to regard *HcPCNA1* as a potential regulatory factor associated with height variation in polyploid plants, whose altered expression may represent one component of the broader reorganization of developmental regulatory networks following changes in genome ploidy.

In addition, transcriptome analysis of TRV-*HcPCNA1* plants identified a total of 12 differentially expressed transcription factors, primarily belonging to the ARF, bZIP, and EIL families. Previous studies have demonstrated that these classes of transcription factors play crucial roles in regulating plant architecture and growth. For example, Lv et al. [48] reported that overexpression of the *MdARF3* gene in apple and tobacco significantly promotes shoot growth and root elongation, thereby increasing plant height. In wheat, mutation of *TaARF12* results in reduced plant height and increased spike length [49]. In rice, overexpression of *OsbZIP61* leads to a pronounced dwarf phenotype accompanied by decreased grain yield [50]. In cotton, the HD-Zip transcription factor *GhHB12* regulates the spatiotemporal distribution, transport, and signaling of auxin, thereby influencing the expression of genes associated with cell wall expansion and ultimately suppressing plant height [51]. Collectively, these findings indicate that transcription factors from different families can directly or indirectly modulate plant height through diverse regulatory pathways. In the present study, silencing of *HcPCNA1* via virus-induced gene silencing caused most of these transcription factors to be downregulated. This observation suggests that ARF, bZIP, and EIL transcription factors may be functionally associated with the transcriptional activity of *HcPCNA1*, jointly contributing to height regulation in *Hedychium*. In other words, suppression of *HcPCNA1* may disrupt the normal growth-regulatory network, resulting in reduced expression of key height-related transcription factors and consequently affecting plant morphology. To further elucidate this regulatory relationship, future studies will employ yeast one-hybrid assays, dual-luciferase reporter analyses, and EMSA to determine whether ARF, bZIP, and EIL proteins directly or indirectly regulate *HcPCNA1* expression, thereby providing stronger evidence for uncovering the molecular mechanisms underlying height regulation in *Hedychium*.

Dwarfism is an important agronomic trait, as shorter crops exhibit improved lodging resistance and allow for higher planting densities, making this trait highly desirable in crop breeding programs in China [52]. Similarly, in ornamental plants, dwarf varieties are particularly suitable for pot cultivation and other horticultural applications, highlighting their potential as key targets in breeding programs. In this study, colchicine was applied to tetraploid *H. coccineum* to induce polyploidy. The resulting mutant plants were successfully doubled to octoploids. Compared with tetraploid plants, the octoploid individuals exhibited reduced growth rates, pronounced dwarfism, fewer leaves per stem, significantly shortened internodes, lighter leaf color, increased leaf angles, and enhanced stress tolerance. Tetraploid *H. coccineum* plants exhibit relatively tall stature, which is unsuitable for potted cultivation. In this study, octoploid dwarf plants were generated via colchicine-induced chromosome doubling, and their phenotypic and dwarfing traits were systematically analyzed. This work provides a theoretical basis for elucidating the mechanisms underlying polyploid-induced dwarfism in plants.

## Figures and Tables

**Table 1:** Interaction effects of colchicine concentration and treatment duration on the growth of primary calli in *Hedychium coccineum*.

Treatment Concentration (g/L)	Treatment Time (d)	Fold Increase	Survival Rate (%)
0	0	8.02 ± 0.28 a	90.00 ± 3.26 a
0.05	3	5.85 ± 0.25 b	75.00 ± 3.38 b
	4	4.76 ± 0.46 c	65.00 ± 3.32 c
	5	3.83 ± 0.32 d	37.50 ± 2.24 e
0.1	3	3.49 ± 0.24 e	60.00 ± 3.26 c
	4	2.33 ± 0.23 f	45.00 ± 2.21 d
	5	1.24 ± 0.27 g	17.50 ± 1.18 g
0.2	3	0.96 ± 0.14 h	27.50 ± 2.21 f
	4	0.53 ± 0.10 h	12.50 ± 1.12 g
	5	0.00 ± 0.00 h	0.00 ± 0.00 h

Note: Different lowercase letters indicate significant differences among treatments at *p* < 0.05.

**Table 2:** Comparison of phenotypic traits between tetraploid and octoploid *H. coccineum* during the flowering stage.

Traits	Tetraploid	Octoploid
Vegetative growth period (months)	4~5	4~5
Bud break period (months)	2~3	2~3
Dormancy period (months)	December to February of the following year	Almost no dormancy
Flowering period (months)	June to October	Mid-May to early June
Flowering period of an inflorescence (day)	8~10	2~3
Flowering period of a single flower (day)	2~3	1.5~2.5
Leaf emergence rate (days per leaf)	3.68	4.90
Plant height (cm)	183.09 ± 39.24 a	72.98 ± 6.30 b
Plant spread (m^2^)	1.95 ± 0.67 a	0.30 ± 0.11 b
Number of tillers(branches)	29.89 ± 11.31 a	20.60 ± 7.40 a
Number of internodes (number)	15.78 ± 2.99 a	10.5 ± 0.58 b
Pseudostem diameter (cm)	16.25 ± 2.42 a	16.65 ± 0.78 a
Average internode length (cm)	11.99 ± 3.60 a	6.95 ± 0.38 b
Leaf length (cm)	42.58 ± 3.34 a	20.38 ± 1.11 b
Leaf width (cm)	5.01 ± 0.42 a	3.63 ± 0.29 b
Leaf angle (°)	55.36 ± 4.03 b	71.67 ± 5.17 a
Stomata Density (no./mm^2^)	75.43 ± 2.23 a	36.84 ± 1.72 b
Stomata Length (μm)	32.20 ± 1.86 a	52.37 ± 4.71 b
Stomata Width (μm)	30.52 ± 2.88 a	78.30 ± 8.01 b
Inflorescence length (cm)	25.38 ± 5.31 a	8.15 ± 0.26 b
Inflorescence width (cm)	14.13 ± 1.84 a	4.95 ± 0.13 b
Number of bracteoles (number)	42.75 ± 2.22 a	19.00 ± 1.73 b
Flower length (cm)	5.43 ± 0.10 a	4.58 ± 0.17 b
Flower width (cm)	3.75 ± 0.17 a	2.93 ± 0.19 b
Bract length (cm)	3.65 ± 0.31 a	2.85 ± 0.19 b
Bract width (cm)	1.38 ± 0.25 a	1.29 ± 0.09 a
Bracteole length (cm)	1.98 ± 0.21 a	1.76 ± 0.05 a
Bracteole width (cm)	1.14 ± 0.15 a	1.16 ± 0.11 a
Calyx length (cm)	2.89 ± 0.14 a	2.65 ± 0.25 a
Calyx width (cm)	0.60 ± 0.07 b	0.80 ± 0.04 a
Corolla tube length (cm)	3.03 ± 0.10 a	1.65 ± 0.12 b
Corolla tube width (cm)	0.19 ± 0.02 b	0.33 ± 0.03 a
Lobe length (cm)	3.15 ± 0.27 a	2.93 ± 0.05 a
Lobe width (cm)	0.23 ± 0.03 a	0.32 ± 0.05 a
lip petal length (cm)	2.48 ± 0.19 a	2.84 ± 0.17 a
lip petal width (cm)	1.85 ± 0.18 a	1.76 ± 0.09 a
Lateral petal length (cm)	2.60 ± 0.16 a	2.88 ± 0.10 a
Lateral petal width (cm)	0.43 ± 0.06 a	0.29 ± 0.03 b
Pistil length (cm)	8.00 ± 0.08 a	5.68 ± 0.17 b
Pistil width (cm)	0.19 ± 0.02 b	0.33 ± 0.03 a
Stamen length (cm)	5.16 ± 0.11 a	3.91 ± 0.09 b
Stamen width (cm)	0.21 ± 0.02 a	0.28 ± 0.03 a
Anther length (cm)	0.73 ± 0.09 b	0.91 ± 0.03 a
Anther width (cm)	0.21 ± 0.02 a	0.28 ± 0.03 a
Filament length (cm)	4.45 ± 0.07 a	2.90 ± 0.08 b
Filament width (cm)	0.14 ± 0.02 b	0.21 ± 0.02 a

Note: Different lowercase letters indicate significant differences (*p* < 0.05) in the same trait between tetraploid and octoploid *H. coccineum.*

**Table 3:** Statistics of sequencing data.

Sample	Clean Reads	Clean Bases	GC Content	Q30	Total Mapping Reads
T1	39,578,990	7,994,174,796	49.42%	94.46%	68.99%
T2	38,755,407	7,827,821,973	49.31%	94.61%	68.89%

## Data Availability

The data that supports the findings of this study are available in the Supplementary Material of this article.

## Supplementary Materials

The following supporting information can be downloaded at: https://www.mdpi.com/article/10.3390/plants14233573/s1, Figure S1: Changes in callus morphology of *H. coccineum* under different colchicine concentrations. (A) Colchicine concentration of 0.05 g/L; (B) Colchicine concentration of 0.1 g/L; (C) Colchicine concentration of 0.2 g/L; Figure S2: Growth status of tetraploid and octoploid *H. coccineum* seedlings. (A) The growth status of tetraploid and octaploid *H. coccineum* in tissue culture bottles; (B) The growth status of tetraploid and octaploid *H. coccineum* under outdoor conditions; Table S1: Primer sequences for gene cloning, vector construction, and qRT-PCR; Table S2: Ploidy analysis of colchicine-induced *H. coccineum* plants.
