# Supplementary material for: Induction of Autopolyploidy and Preliminary Investigation of the Dwarfing Mechanism in Hedychium coccineum
Source: Plants (Basel). 2025 Nov 22;14(23):3573. doi: 10.3390/plants14233573 (PMC12693814; doi:10.3390/plants14233573)
Supplement: Supplementary file 1 [file plants-14-03573-s001.zip › plants-3975569-supplementary.pdf]

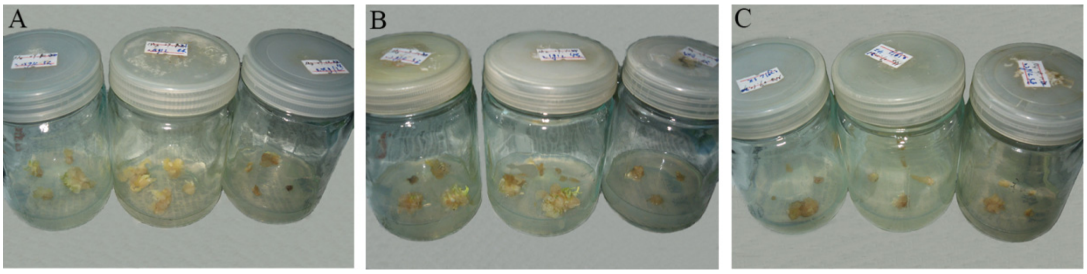

Figure S1 Changes in callus morphology of *Hedychium coccineum* under different colchicine concentrations. **(A)** Colchicine concentration of 0.05 g/L; **(B)** Colchicine concentration of 0.1 g/L; **(C)** Colchicine concentration of 0.2 g/L.

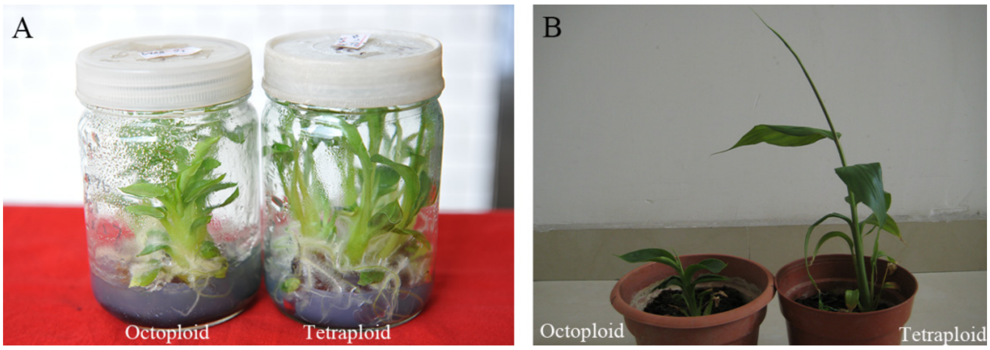

Figure S2 Growth status of tetraploid and octoploid *H. coccineum* seedlings. (A) The growth status of tetraploid and octoploid *H. coccineum* in tissue culture bottles; (B) The growth status of tetraploid and octoploid *H. coccineum* under outdoor conditions.

Table S1 Primer sequences for gene cloning, vector construction, and qRT-PCR

Name	Primer Sequence（5'-3'）
qPCR-HcPCNA1-F	AACTTCGACTGCTCCCGC
qPCR-HcPCNA1-R	GATGGATCGGCTGCGGTC
HcGAPDH-F	TAACATCATTCCCAGCAGCACTG
HcGAPDH-R	GAGCCTGACAGTGAGATCCAC
AtActin-F	GCAGAGCGGGAAATTGTAAG
AtActin-R	GTACAGATCCTTCCTGATATCC
HcPCNA1-F	ATGCGGGAGCTGGTCACCGACGCTA
HcPCNA1-R	TCATTCATTTCGATTATCATGGATTC
pOx-HcPCNA1-F	gggtaccggcgcgccaagcttATGCGGGAGCTGGTCACC
pOx-HcPCNA1-R	caattcacacttgtaggatccTTCATTTCGATTATCATGGATTCC
pTRV2-HcPCNA1-F	gtgagtaaggttaccgaattcATGCGGGAGCTGGTCACC

pTRV2-HcPCNA1-R	cgtgagctcggtaccggatccTTCATTTCGATTATCATGGATTCC

Table S2 Ploidy analysis of colchicine-induced *H. coccineum* plants

Colchicine-induced *H. coccineum* plant	Mean fluorescence intensity ratio (colchicine-induced/tetraploid type)	CV (colchicine-induced)
01	1.83±0.02	5.58±3.13%
02	1.89±0.07	3.50±0.73%
03	2.20±0.07	6.80±1.79%
04	1.78±0.11	3.04±0.11%
05	2.15±0.07	4.70±0.40%
06	1.71±0.03	4.14±1.29%
07	1.83±0.08	3.51±0.06%
08	1.84±0.09	1.95±1.48%
09	1.82±0.09	5.21±0.37%
10	1.90±0.01	3.41±0.21%
